# Dexamethasone and Monophosphoryl Lipid A Induce a Distinctive Profile on Monocyte-Derived Dendritic Cells through Transcriptional Modulation of Genes Associated With Essential Processes of the Immune Response

**DOI:** 10.3389/fimmu.2017.01350

**Published:** 2017-10-23

**Authors:** Paulina A. García-González, Katina Schinnerling, Alejandro Sepúlveda-Gutiérrez, Jaxaira Maggi, Ahmed M. Mehdi, Hendrik J. Nel, Bárbara Pesce, Milton L. Larrondo, Octavio Aravena, María C. Molina, Diego Catalán, Ranjeny Thomas, Ricardo A. Verdugo, Juan C. Aguillón

**Affiliations:** ^1^Programa Disciplinario de Inmunología, Facultad de Medicina, Instituto de Ciencias Biomédicas (ICBM), Universidad de Chile, Santiago, Chile; ^2^Millennium Institute on Immunology and Immunotherapy, Santiago, Chile; ^3^Programa de Genética Humana, Facultad de Medicina, Instituto de Ciencias Biomédicas (ICBM), Universidad de Chile, Santiago, Chile; ^4^Translational Research Institute, University of Queensland Diamantina Institute, Woolloongabba, QLD, Australia; ^5^Banco de Sangre, Hospital Clínico de la Universidad de Chile, Santiago, Chile

**Keywords:** tolerogenic dendritic cells, immune regulation, dexamethasone, transcriptome, tolerance induction

## Abstract

There is growing interest in the use of tolerogenic dendritic cells (tolDCs) as a potential target for immunotherapy. However, the molecular bases that drive the differentiation of monocyte-derived DCs (moDCs) toward a tolerogenic state are still poorly understood. Here, we studied the transcriptional profile of moDCs from healthy subjects, modulated with dexamethasone (Dex) and activated with monophosphoryl lipid A (MPLA), referred to as Dex-modulated and MPLA-activated DCs (DM-DCs), as an approach to identify molecular regulators and pathways associated with the induction of tolerogenic properties in tolDCs. We found that DM-DCs exhibit a distinctive transcriptional profile compared to untreated (DCs) and MPLA-matured DCs. Differentially expressed genes downregulated by DM included MMP12, CD1c, IL-1B, and FCER1A involved in DC maturation/inflammation and genes upregulated by DM included JAG1, MERTK, IL-10, and IDO1 involved in tolerance. Genes related to chemotactic responses, cell-to-cell signaling and interaction, fatty acid oxidation, metal homeostasis, and free radical scavenging were strongly enriched, predicting the activation of alternative metabolic processes than those driven by counterpart DCs. Furthermore, we identified a set of genes that were regulated exclusively by the combined action of Dex and MPLA, which are mainly involved in the control of zinc homeostasis and reactive oxygen species production. These data further support the important role of metabolic processes on the control of the DC-driven regulatory immune response. Thus, Dex and MPLA treatments modify gene expression in moDCs by inducing a particular transcriptional profile characterized by the activation of tolerance-associated genes and suppression of the expression of inflammatory genes, conferring the potential to exert regulatory functions and immune response modulation.

## Introduction

Dendritic cells (DCs) are a heterogeneous group of specialized antigen-presenting cells with the capacity to orchestrate specific immune responses according to the antigen they encounter and the environmental signals derived from the local milieu (1). After antigen capture and processing, DCs undergo a complex process of differentiation to mature DCs, which express high levels of surface peptide–HLA complexes and co-stimulatory molecules. Mature DCs also produce inflammatory cytokines and T-cell-attracting chemokines enabling the induction of Th1, Th2 or Th17 responses (2, 3). DCs can also differentiate into tolerogenic DCs (tolDCs), capable of inducing anergy or deletion of effector T cells, and/or differentiation and proliferation of regulatory T-cell (Treg) subsets. Regulation may result from various processes, including deficient antigen presentation, reduced co-stimulatory molecules, expression of inhibitory molecules, and/or secretion of anti-inflammatory cytokines such as IL-10 and TGF-β (4, 5). The ability of DCs to modulate T-cell responses has made them an interesting target of study for the immunotherapy of autoimmune diseases, since these cells are supposed to induce and maintain immune tolerance to harmless or self-antigens (6, 7).

Differentiation of DCs from peripheral blood monocytes using GM-CSF and IL-4 is a useful approach to obtain large numbers of DCs *in vitro* to study their function and biology. This approach is also used to generate tolDCs *in vitro* by adding immune modulators such as immunosuppressant drugs [dexamethasone (Dex), rapamycin, aspirine, rosiglitazone, tacrolimus] (8–12); anti-inflammatory cytokines (IL-10 and TGF-β) (13–15); natural compounds (vitamin D3, retinoic acid, and curcumin) (8, 16, 17); the JAK inhibitor tofacinib (18); and the NF-kB inhibitor BAY11-7082 (19). All strategies lead to DCs with regulatory capacities, although some features may vary between protocols. Our group has described a protocol for tolDC generation from peripheral blood monocytes of healthy controls (20) and rheumatoid arthritis (RA) patients (21) using Dex to induce a tolerogenic phenotype and subsequent activation with the non-toxic lipopolysaccharide (LPS) analog monophosphoryl lipid A (MPLA) to confer lymph node homing capacity and stability. These cells, herein termed Dex-modulated and MPLA-activated DCs (DM-DCs), expressed low levels of CD83, CD86, and CD40, secreted high levels of IL-10 and TGF-β and low levels of IL-12, and stimulated T-cell proliferation and cytokine production at low levels in allogeneic and autologous cultures (20, 22).

While we generally understand the cellular mechanisms by which tolDCs modulate T-cell responses and induce tolerance, the molecular switches determining tolDC differentiation and function are still poorly known. The knowledge of molecular regulators and pathways could be of great benefit for searching targets for effective cellular therapies. However, only few studies have attempted to identify specific molecules or processes involved in tolerogenic functions of monocyte-derived DCs (moDCs) using whole-genome transcriptomic or proteomic analyses (23, 24). Most studies focus on vitamin D3-modulated moDCs (25, 26). Studies of Dex-treated moDCs comprised only proteomic approaches and focused on the identification of potential tolDC markers (27, 28).

We recently compared tolDCs derived from monocytes of healthy controls and RA patients at phenotypic, functional, and transcriptional levels (21) and demonstrated that Dex and MPLA treatments removed disease-associated features of moDCs to yield a uniform signature. Here, we describe a genome-wide differential expression study of tolDCs derived from monocytes of healthy controls in which we elucidate molecular processes that drive DC differentiation toward a tolerogenic profile in response to Dex and MPLA treatments. We found that DM-DCs exhibit a transcriptional profile that distinguishes them from other moDC subsets, characterized by the upregulation of several genes related to immunoregulatory functions and biological processes that could be involved in tolerance induction.

## Materials and Methods

### Blood Samples

A total of 10 buffy coat samples from healthy controls were used for microarray analysis and phenotypic and functional studies. An additional 10 buffy coat samples were used to confirm differential expression of genes by qRT-PCR and flow cytometry. All subjects signed an informed written consent and all procedures were approved by the Ethics Committees for Research in Human Beings from the Faculty of Medicine and from the Clinical Hospital of the University of Chile. Demographic characterization of healthy controls is detailed in Table S1 in Supplementary Material.

### Generation of moDC Subsets

Human moDCs were generated from monocytes as previously described (20). Monocytes were isolated from peripheral blood of 10 healthy individuals by negative selection using RosetteSep Human Monocytes enrichment cocktail (Stemcell Technologies, Vancouver, BC, Canada) according to manufacturer’s instructions. Monocytes were cultured at 2 × 10^6^ cells/ml in serum-free AIM-V medium (Gibco BLR, Grand Island, NY, USA), supplemented with 500 U/ml of recombinant human GM-CSF and IL-4 (eBioscience, San Diego, CA, USA) for 5 days at 37°C and 5% CO_2_. At day 3, culture medium was replenished and cells were incubated with Dex (Sigma-Aldrich, St. Louis, CO, USA) at a final concentration of 1 µM [Dex-modulated DCs (D-DCs)]. At day 4, cells were stimulated with 1 µg/ml of cGMP-grade MPLA (Avanti Polar Lipids Inc., Alabaster, AL, USA) (DM-DCs). Unstimulated cells (DCs) and MPLA-matured DCs (M-DCs) generated in the absence of Dex were used as controls of immature and mature DCs, respectively. On day 5, cells were harvested and characterized by flow cytometry.

### Flow Cytometry

Antibodies used for analysis were anti-human CD80 FITC (clone 2D10.4), CD83 FITC (clone HB15e), CD40 PE (clone 5C3), CD86 PE (clone IT2.2), IDO1 PECy7 (clone eyedio), CD4 PECy7 (clone OKT4), IFN-γ APC (clone 4S.B3) (eBioscience); CD11c BUV395 (clone B-ly6), CD83 BUV737 (clone HB15e) (BD Biosciences); CD86 BV650 (IT2.2), CD163 BV605 (clone GHI/61), CD1c BV510 (clone L161), MERTK BV421 (clone 590H11G1E3), CD32 APC (clone FUN-2), ZBTB16/PLZF PE (clone Mags.21F7) (BioLegend) and TLR2 Alexa Fluor 700 (clone 383936), JAG1 Fluorescein (clone 188331), and FPR2 APC (clone 304405) (R&D Systems). Prior to antibody staining, cells were labeled with fixable viability dye eFluor 780 (eBioscience). Cells were resuspended in PBS supplemented with 10% of fetal bovine serum (FBS) (HyClone Thermo Scientific, Logan, UT, USA), stained with specific antibodies, fixed with IC fixation buffer (eBioscience), and resuspended in FACSFlow buffer (Becton Dickinson, San Diego, CA, USA) for subsequent analysis. Data were acquired on a FACSAria III with FACSDiva v6.1.3 software (both Becton Dickinson) and analyzed by FlowJo software (Treestar, USA).

### Cytokine Production

A total of 1 × 10^5^ DCs were incubated for 24 hours with or without CD40L-transfected irradiated NIH3T3 cells at 1:1 ratio in AIM-V medium, in 96-well U bottom plates (BRAND, Wertheim, Germany). Supernatants of cocultures with NIH3T3 cells or T cells were recovered and stored at −80°C until quantification of IL-10, IL-12p70, and IFN-γ by ELISA (eBioscience).

### CD4^+^ T Cell-Stimulatory Capacity of DCs

CD4^+^ T cells were isolated by negative selection using RosetteSep Human T-cell enrichment cocktail (Stemcell Technologies) and labeled with carboxyfluorescein diacetate succinimidyl ester (CFSE).

For the assessment of antigen-specific CD4^+^ T-cell activation, DCs were loaded with 1 µg/ml tuberculin purified protein derivative (Staten Serum Institute, Copenhagen, Denmark) 4 hours prior to activation with MPLA and co-cultured with autologous CD4^+^ T cells at a DC:T-cell ratio of 1:2 in RPMI medium (HyClone Thermo Scientific) with 10% FBS in 96-well U bottom plates for 6 days (20). CD4^+^ T cells alone and stimulated with anti-human CD3 mAb (clone OKT3; 0.65 μg) (eBioscience) were used as negative and positive controls, respectively. Supernatants were collected to assess cytokine secretion. For intracellular IFN-γ detection, 50 ng/ml phorbol-12-myriastate-13-acetate (Sigma-Aldrich), 1 µg/ml ionomycin (Sigma-Aldrich), and 1 µg/ml brefeldin-A (eBioscience) were added for the last 5 hours of culture. Proliferation and IFN-γ production of CD4^+^ T cells were analyzed by flow cytometry.

### RNA Isolation and Microarray Analysis

RNA was isolated from 5 × 10^5^ DCs on day 5 using total RNA isolation RNeasy Mini Kit (Qiagen, Hilden, Germany) following the manufacturer’s instructions. Yield and quality of RNA samples were evaluated with NanoDrop 1000 spectrophotometer (Thermo Scientific, Waltham, MA, USA) and RNA integrity (RIN score) was analyzed with Agilent 2100 Bioanalyzer (Agilent Technologies, Santa Clara, CA, USA) or LabChip GX/GX II (Caliper LifeSciences, Hopkinton, MA, USA). A total of 40 samples, corresponding to 10 healthy donors under four experimental conditions were considered for microarray analysis (Figure S1 in Supplementary Material). All RNA samples used for microarrays showed A260/A280 values between 1.8 and 2.2, and RIN scores >7. RNA samples were reverse transcribed, amplified, and labeled using an Illumina^®^ TotalPrep™ RNA Amplification Kit, and cDNA was hybridized onto Illumina Human HT-12 v4 BeadChips (Illumina, San Diego, CA, USA), covering the whole human genome. Expression data were extracted with GenomeStudio Project Software from Illumina.

### Confirmation of Gene Expression by qRT-PCR

cDNA was prepared from moDCs RNA samples using the Superscript II Reverse Transcriptase kit (Thermo Fisher Scientific). Quantitative RT-PCR was performed in Stratagene Mx300P, using Brilliant II SYBR Green QPCR Master Mix (Agilent Genomics) with primer sets from IDT. The housekeeping genes *GAPDH* and *r18S* were used as internal controls and target gene expression was normalized to untreated DCs. Primer sequences for each target gene are described in Table S2 in Supplementary Material.

### Data Exploration and Statistical Analyses

For flow cytometry and qPCR data, Friedman repeated measures test and Dunn’s *post hoc* test were used for data comparison between moDC culture conditions. Analyses were performed using Prism 5.01 software (Graphpad, San Diego, CA, USA).

Microarray data were log transformed followed by quantile normalization using the preprocess Core package v1.28.0 from Bioconductor. Differentially expressed (DE) genes in modulated DCs relative to unstimulated DCs were identified with the Maanova package v1.36.0 *t*-test for gene pairwise comparisons (29), and *p*-values were adjusted using false discovery rate (FDR) method. Genes with adjusted *p*-value ≤0.05 were considered differentially expressed and reported. K-means clustering of residual values of DE genes between DM-DCs and DCs was performed using the cluster package and a *K* value of 6 to maximize cluster distance and minimize distance between clustered genes.

Overrepresentation of pathways and biological functions was assessed using Ingenuity Pathway Analysis (Ingenuity Systems, Qiagen, Hilden, Germany).

## Results

### Modulation of moDCs with Dex and MPLA Induces a Distinctive Transcriptional Profile

**Figure 1:**
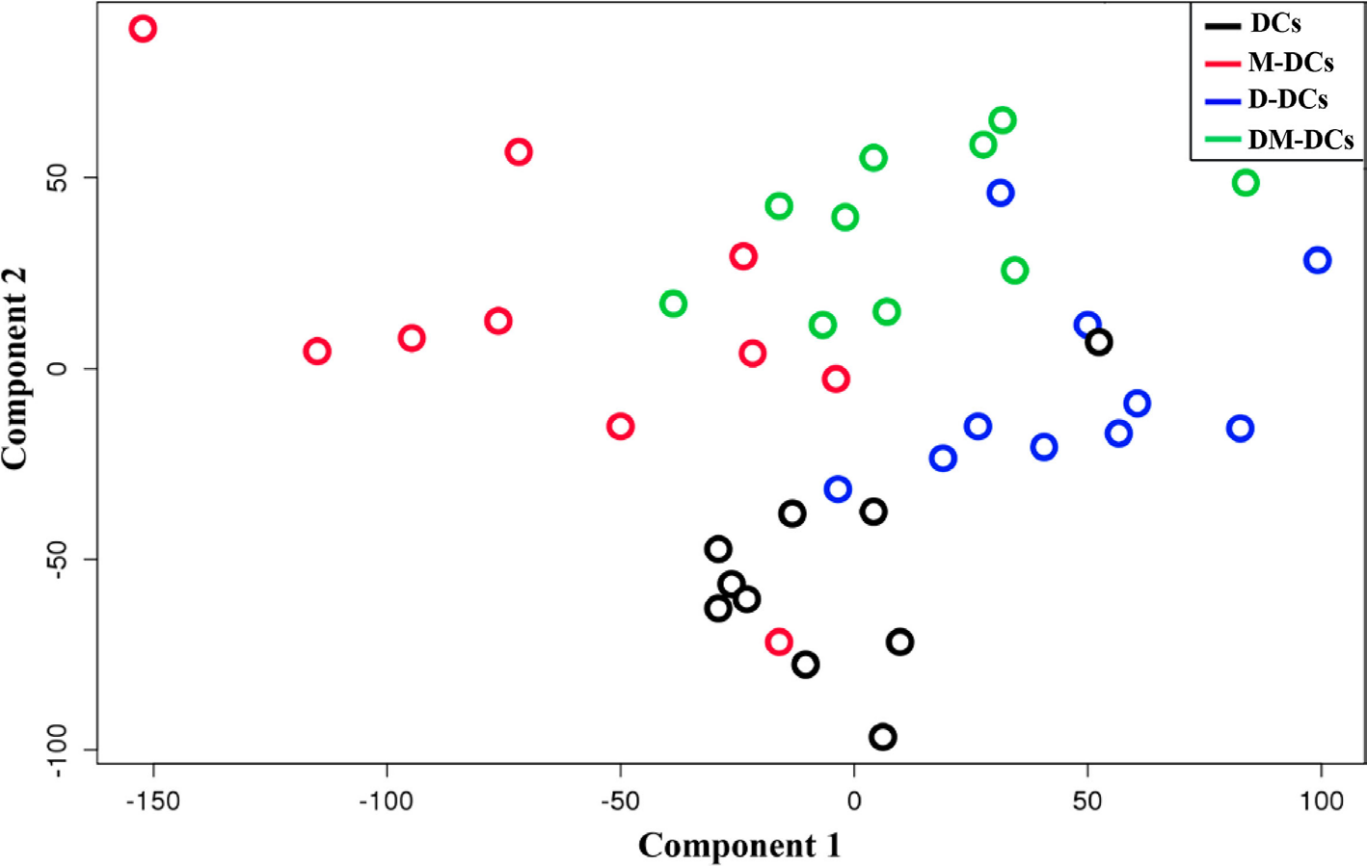
Different stimuli used for monocyte-derived DCs (moDCs) differentiation induces a particular transcriptional profile that distinguishes them from other subtypes. Principal component analysis of the two first components allows separation of all four moDCs experimental groups according to variance values.

First, we confirmed that treatment with Dex and MPLA during moDC differentiation induces a tolerogenic phenotype on these cells. As previously described (20), DM-DCs expressed low levels of CD86, CD80, CD40, and CD83 and produced low levels of IL-12 and high levels of IL-10 relative to M-DCs. T-cell proliferation and IFN-γ production in response to antigen-exposed DM-DCs were significantly reduced when compared with T cells stimulated with DCs or M-DCs (Figure S2 in Supplementary Material). Subsequently, we used RNA from the same moDC preparations to conduct whole-genome analysis through microarray technology, using a total of 40 moDC samples, corresponding to four different DC subsets, i.e., unstimulated (DC), M-DCs, D-DCs, and Dex-modulated/MPLA-activated DCs (DM-DC), differentiated from monocytes from 10 healthy individuals. We defined statistically significant differences between samples by a FDR value of 0.05 or lower. Since all four differentiation protocols led to moDCs with different phenotypic characteristics, we tested whether these differences could also be found at transcriptional level. A principal component analysis was used to explore the data by projecting samples onto the major orthogonal components or genes expression. The first two dimensions separated moDCs samples by their differentiation state (unstimulated, MPLA, Dex, or Dex plus MPLA) (Figure 1). The first component (*X*-axis) naturally clustered samples as M-DCs < DCs < (D-DCs:DM-DCs). D-DCs and DM-DCs, which together could be associated with a tolerogenic potential, could not be distinguished on component 1. The second component (*Y*-axis) clustered samples by their grade of activation, with both moDC subtypes treated with MPLA projecting toward positive values, i.e., DCs < D-DCs < (M-DCs:DM-DCs). Thus, each protocol induces a unique transcriptional profile in moDCs that distinguishes them from other moDC subtypes.

### Dex and MPLA Treatment Modulates Genes Associated With Cell Movement, Signaling, and Metabolism

Given their tolerogenic phenotype, we next focused on the transcriptional effects of combined treatment with Dex and MPLA treatment on moDCs. Considering *p*-values (corrected using FDR) 0.05 or lower to define statistically significant differences in gene expression, we identified 259 DE transcripts in DM-DCs compared to the unstimulated control (DCs). The scavenger receptor CD163, several MT1 (metallothionein 1) isoforms and MT2A, C1QTNF1, ADORA3, S100A9, and both isoforms of Fc receptor for IgG FCGR2A/CD32 and FCGR2B/CD32B (Table 1; Table S3 in Supplementary Material) were among the genes most upregulated by Dex and MPLA treatments; while CD1b, FCER1A (high affinity I Fc fragment of IgE receptor subunit alpha polypeptide), and MMP12 (Matrix metallopeptidase 12) were the most downregulated genes (Table 1; Table S3 in Supplementary Material).

**Table 1:** Top 20 of most regulated genes by treatment of monocyte-derived dendritic cells with dexamethasone and monophosphoryl lipid A.

Gene ID	Gene name	Fold change
CD163	CD163 molecule	4.83
MT1G	Metallothionein 1G	4.35
MT1H	Metallothionein 1H	4.23
MT1E	Metallothionein 1E	3.12
C1QTNF1	C1q and tumor necrosis factor-related protein 1	2.99
MT2A	Metallothionein 2A	2.97
MT1M	Metallothionein 1M	2.92
MT1A	Metallothionein 1A	2.63
MAFB	V-Maf Avian musculoaponeurotic fibrosarcoma oncogene homolog B	2.59
MT1X	Metallothionein 1X	2.49
ADORA3	Adenosine A3 receptor	2.45
CD32	Low affinity IIA Fc fragment of IgG receptor	2.43
IFITM3	Interferon-induced transmembrane protein 3	2.39
TSC22D3/GILZ	Glucocorticoid-induced leucine zipper protein	2.33
MT1F	Metallothionein 1F	2.30
S100A9	S100 calcium-binding protein A9	2.28
C1QA	Complement component 1, subcomponent Q, A chain	2.24
FCER1A	High-affinity 1 Fc fragment of IgE receptor subunit alpha polypeptide	−2.45
MMP12	Matrix Metallopeptidase 12	−2.52
CD1B	CD1B molecule	−3.02

**Figure 2:**
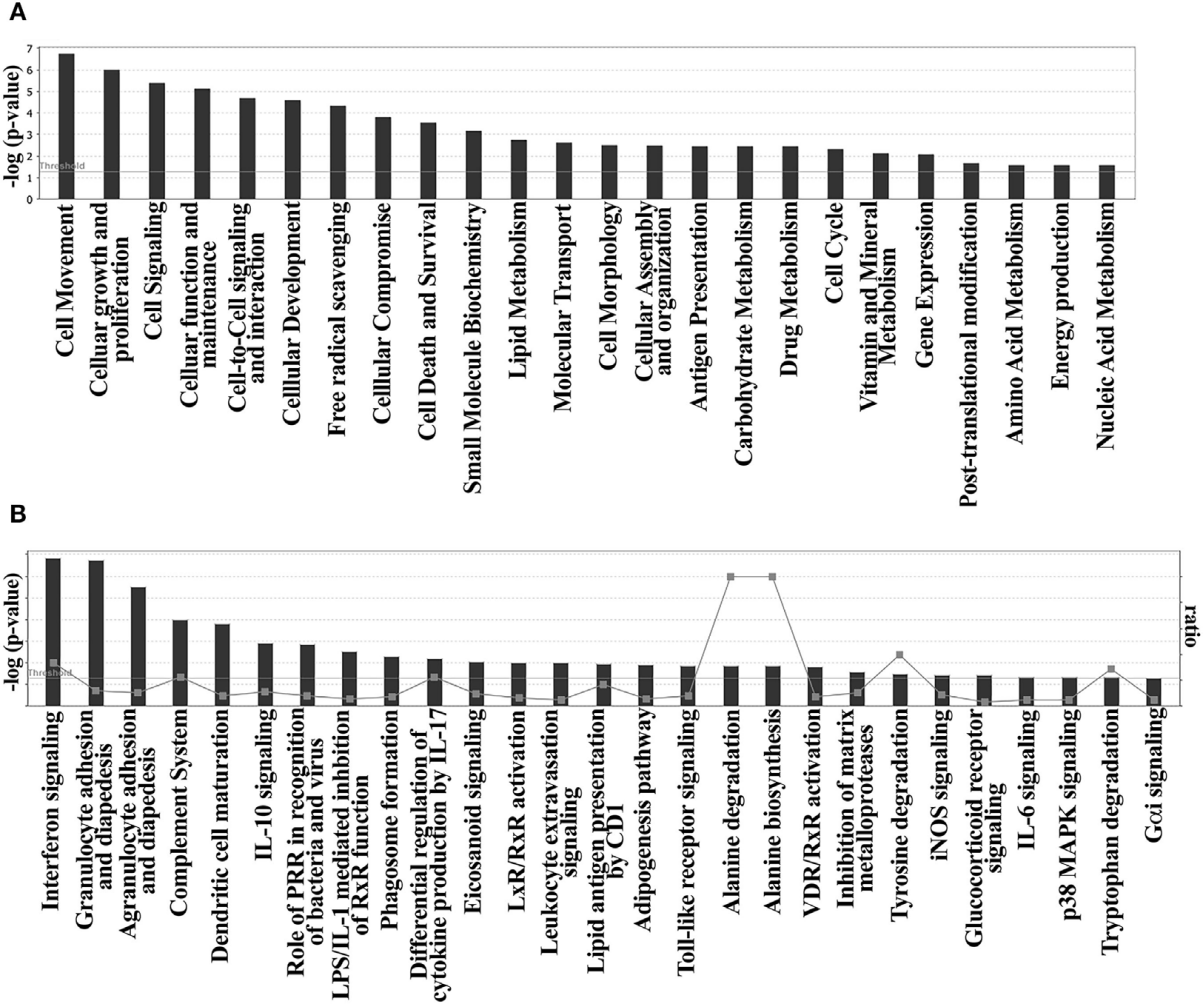
Functional enrichment analysis reveals modulation of cell migration, signaling and metabolism on dexamethasone (Dex)-modulated and monophosphoryl lipid A (MPLA)-activated dendritic cells (DM-DCs). Ingenuity pathway analysis (IPA) functional GO overrepresentation of biological functions of 259 differentially expressed genes found on monocyte-derived DCs shows an enrichment of genes related to cell movement, cellular proliferation, signaling, and metabolism. Consistent with functional analysis, Dex and MPLA were found to modulate genes involved in the canonical pathways of cell signaling, cell adhesion and diapedesis, complement system, DC maturation and metabolism. **(A)** Biological functions enriched on DM-DCs. **(B)** Canonical pathways overrepresented on DM-DCs.

A pathway enrichment analysis of the DE genes found on DM-DCs done with ingenuity pathway analysis (IPA) identified cell movement, cell signaling, and metabolism as main functions modulated by Dex and MPLA (Figure 2A; Table S4 in Supplementary Material), assembling regulated genes into 11 networks representing significantly enriched biological functions, mostly associated with cellular movement, growth, immune cell trafficking, and metabolism (Figure 2A; Table S4 in Supplementary Material). The main regulated genes in DM-DCs such as CD163, ADORA3, FCGR2A, CD1c, CD1b, and MMP12 belong to these networks. In terms of canonical pathways and consistent with functional enrichment analysis, Dex and MPLA treatments affected cell adhesion and diapedesis (*p*-value 8.52e−3) (Figure 2B), modulating the expression of 10 genes, including the upregulation of chemokines such as CCL8, CCL18, CCL23, and CXCL5. Interferon signaling (*p*-value 7.49e−3) was predicted to be an active process, with a positive *z*-score (2.449) and high expression levels of the IFN-inducible genes IFI6, IFITM1, IFITM2, and IFITM3 (Table S4 in Supplementary Material). Processes associated with DC maturation and activation were inhibited, and several molecules associated with these processes such as CD80, CD83, CD1c, ACTA2, ACTG1, TMBS10, AP-1, and RAP1GAP were downregulated (Table S4 in Supplementary Material). Complement system pathway was highly modulated, with increased expression of C1QA, C1QB, C1QC, and CFB. IL-10 signaling (*p*-value 2.93e−3), along with TLR (*p*-value 4.11e−2), iNOS (*p*-value 4.65e−2), and p38MAPK signaling (*p*-value 1.31e−2), which also signal through IRAK and STAT1, was enriched processes on DM-DCs according to IPA knowledge database (Figure 2B; Table S4 in Supplementary Material). Upstream regulator analysis revealed GILZ, STAT1, FOXO3, STAT3, SMARCA4 and CEBPD to be amongst the main transcriptional regulators of many genes from this dataset (Table S4 in Supplementary Material).

### The Transcriptional Program of DM-DCs Is Regulated by an Interplay between Dex and MPLA

**Figure 3:**
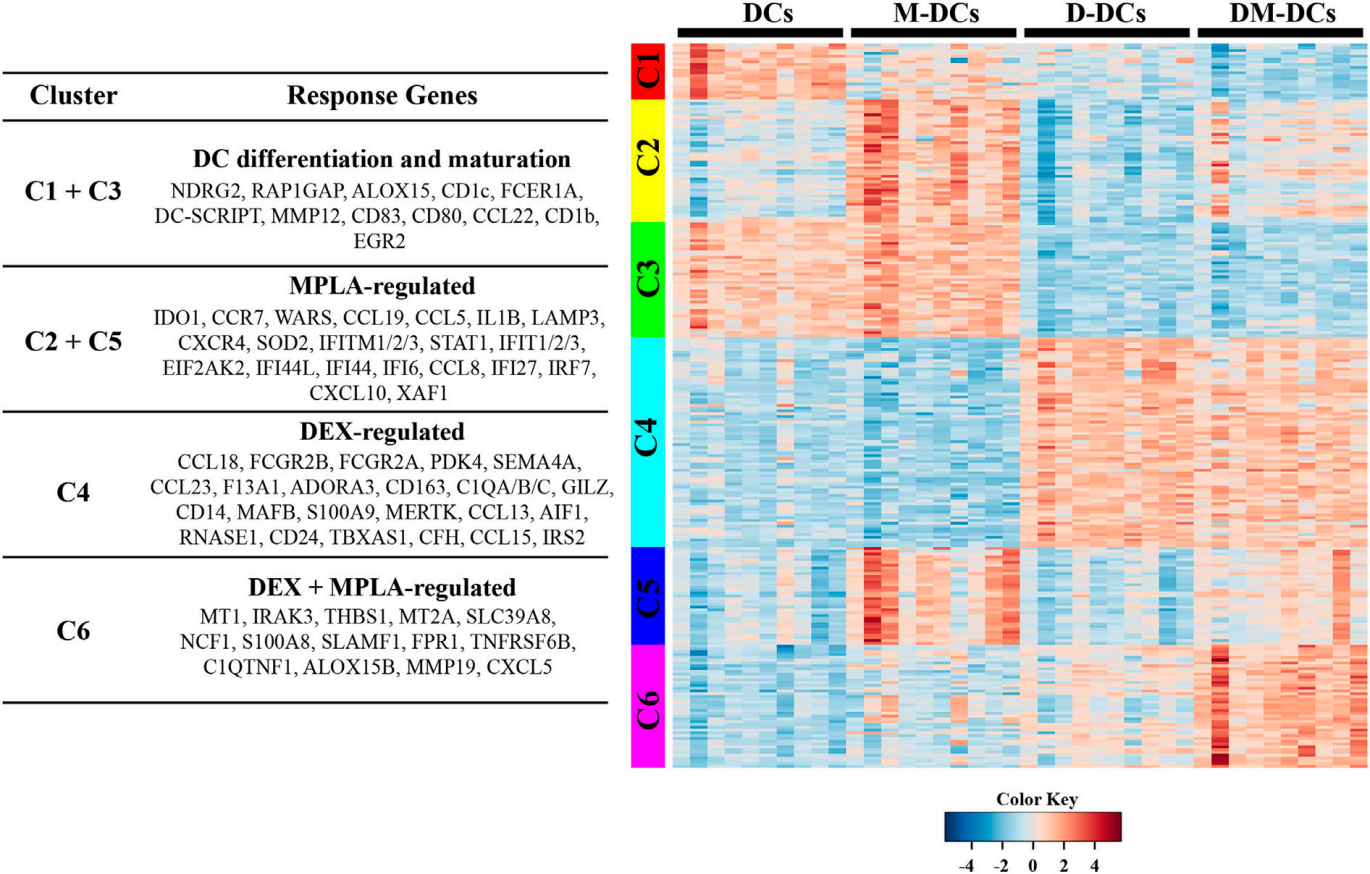
Transcriptional program of dexamethasone (Dex)-modulated and monophosphoryl lipid A (MPLA)-activated dendritic cells (DM-DCs) is the result of gene expression regulation by an interplay between dexamethasone and MPLA. A clustering analysis was performed using a K-means clustering of the residual values of the 259 differentially expressed transcripts found on DM-DCs *versus* DCs. Each cluster is represented by a different color band. Clusters that shared a similar inferred response toward stimuli used were further grouped, and representative genes for each group are shown in the left and in Table S3 in Supplementary Material.

The 259 DE genes in DM-DCs *versus* untreated DCs were further grouped into six clusters according to their expression pattern using K-means clustering of residual values (clusters C1–C6; Figure 3). Clusters 1 and 3 contain 61 DE genes (Cluster 1 = 20 genes; Cluster 3 = 41 genes) that correspond mainly to DC differentiation and maturation, including the DC markers CD1c, DC-SCRIPT/ZNF366, co-stimulatory molecule CD80, and other molecules involved in DC maturation and inflammation such as CD83, RAP1GAP, NDRG2, CD1b, FCER1A, CCL22, and MMP12 (Figure 3; Table S3 in Supplementary Material). Dex treatment alone or combined with MPLA leads to downregulation of these genes (Figure 3). Clusters 2 and 5 comprise 79 genes upregulated by MPLA (Cluster 2 = 44 genes; Cluster 5 = 35 genes), regardless of the presence of Dex. These clusters contain most genes related to IFN signaling and granulocyte and agranulocyte adhesion and diapedesis, in addition to IL-1B, STAT1, and IDO1. Cluster 4 contains 75 genes, which are upregulated by Dex irrespective of MPLA addition. This cluster includes many genes associated to inhibition of DC activation and maturation such as FCGR2B, C1Q, and MAFB, as well as genes related to anti-inflammatory responses of DCs leading to IL-10 production (TSC22D3/GILZ and MAP3K8/TPL-2) and modulation of T-cell activation and/or expansion of Treg-cell populations such as JAG1 (Jagged 1), MERTK (receptor tyrosine kinase Mer), TBXAS1, and SEMA4A, thus contributing to the tolerogenic profile of DM-DCs (Figure 3; Table S3 in Supplementary Material). Additionally, Cluster 4 is characterized by the expression of membrane receptors (MERTK, FCGR2A, FCGR2B, and SEMA4A), signaling proteins (MAP3K8, S100A9, and JAG1) and transcriptional regulators (TSC22D3/GILZ) as well as molecules related to the complement system (C1QA, C1QB, C1QC, and CFH) and cell adhesion and migration (CCL13, CCL18, CCL23, SH3PXD2B, and ADORA3) (Figure 3; Table S3 in Supplementary Material). IL-10 signaling and complement system are among the pathways overrepresented in this group.

Finally, Cluster 6 contains 44 genes, which were regulated in response to a synergistic effect between Dex and MPLA. Metallothioneins, proteins involved in stress response, heavy metal scavenging, and immunosuppression were highly represented in this group, including several MT1 isoforms (MT1A, MTE, MT1F, MT1G, MT1H, MT1M, and MT1X) and MT2A. Enrichment of several molecules involved in cell adhesion and diapedesis (CXCL5 and MMP19), T-cell migration (THBS1 and TNFRSF6B), and production of reactive oxygen species (ROS) (FPR1, FPR2, NCF1, and SLAMF1) was also found in this cluster as well as molecules associated with a low inflammatory state (C1QTNF1, SLC39A8/ZIP8 and IRAK3), suggesting that genes in this cluster also contribute to the regulatory features of DM-DCs in addition to the genes of Cluster 4. Functional and pathways analysis of genes from this cluster showed that cellular movement and metabolic processes, particularly ROS metabolism is highly represented (Table S5 in Supplementary Material).

### Dex and MPLA Treatment Promotes the Upregulation of Genes Related to the Modulation of Biological Processes That Control Immune Responses

**Figure 4:**
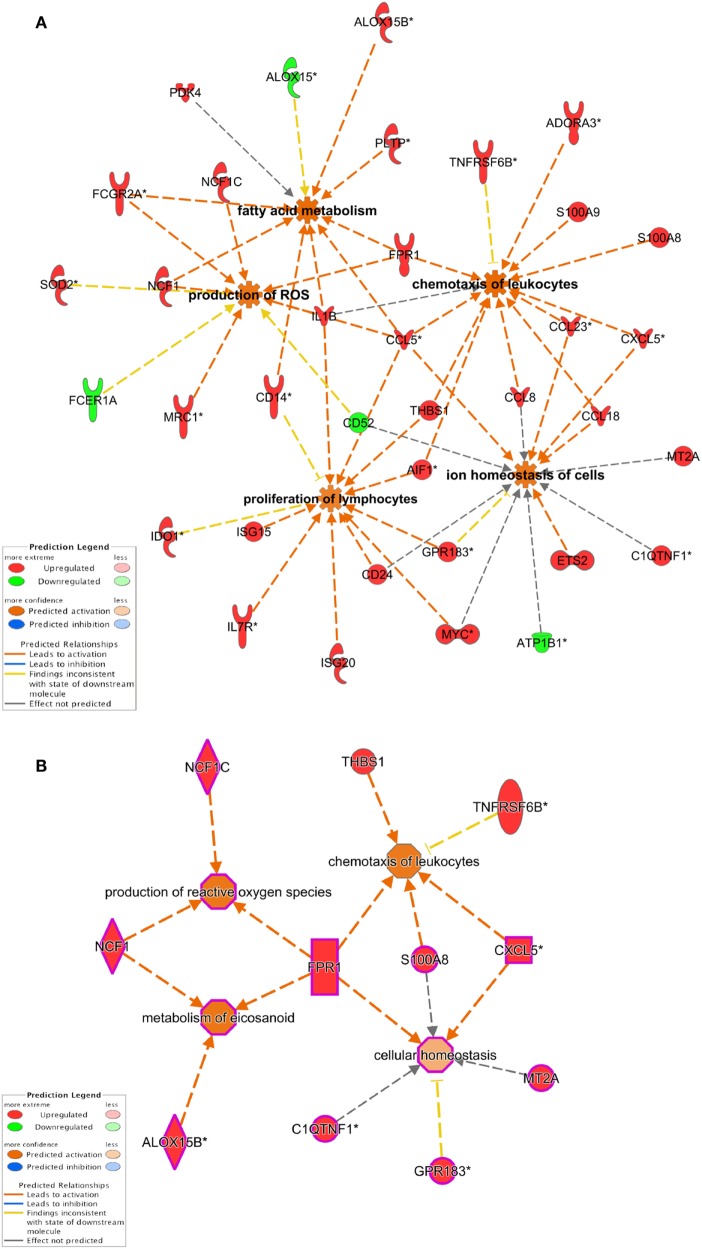
Dexamethasone (Dex) and monphosphoryl lipid A (MPLA) treatment leads to changes in the expression of genes involved in biological processes associated with immune response regulation. **(A)** Ingenuity pathway analysis network interaction analysis of differentially expressed genes in Dex-modulated and MPLA-activated dendritic cells shows that genes modulated by Dex and MPLA are involved in the control of T-cell activation, cell movement, and metabolism. **(B)** Network interaction analysis of genes from Cluster 6 (see Figure 3), modulated synergically by dexamethasone and MPLA.

Biological functions related to cellular movement, growth, and proliferation, cell-to-cell signaling, and free radical scavenging were found to be upregulated. Canonical pathway analysis also provided agranulocyte/granulocyte adhesion and diapedesis, T helper differentiation, and DC maturation as main pathways regulated in our dataset (Figure 2; Tables S4 and S5 in Supplementary Material). Since these functions were highly enriched in the two clusters that are potentially involved in DM-DCs tolerogenic features (Clusters 4 and 6), we further analyzed interactions between genes contained in these clusters. Functional annotations involving activation and proliferation of T lymphocytes were highly represented in several networks and predicted to be inhibited in DM-DC, while chemotaxis of T lymphocytes and synthesis of ROS, also highly represented, were predicted to be activated in these cells (Figure 4). Most downregulated genes in DM-DCs, which are associated with T-cell activation and proliferation, are also involved in DC maturation and activation, while upregulated genes interacting in these networks encode inhibitory membrane receptors or transcriptional regulators that lead to inhibition of effector T-cell activation and promote differentiation of Tregs (FCGR2B, MT1, IDO1, CCL18, JAG1, and MERTK). This is further supported by upregulation of genes involved in the activation of ROS production (FPR1 and NCF1 and NAMPT) and recruitment of naive and Tregs (CCL18, CCL23, and SAA1). Many of the genes that were upregulated in response to Dex and MPLA together (Cluster 6) also participate in the main metabolic processes in DM-DCs, mainly ROS production and zinc homeostasis (Table S5 in Supplementary Material), contributing to the particular metabolic profile of these tolDCs.

**Figure 5:**
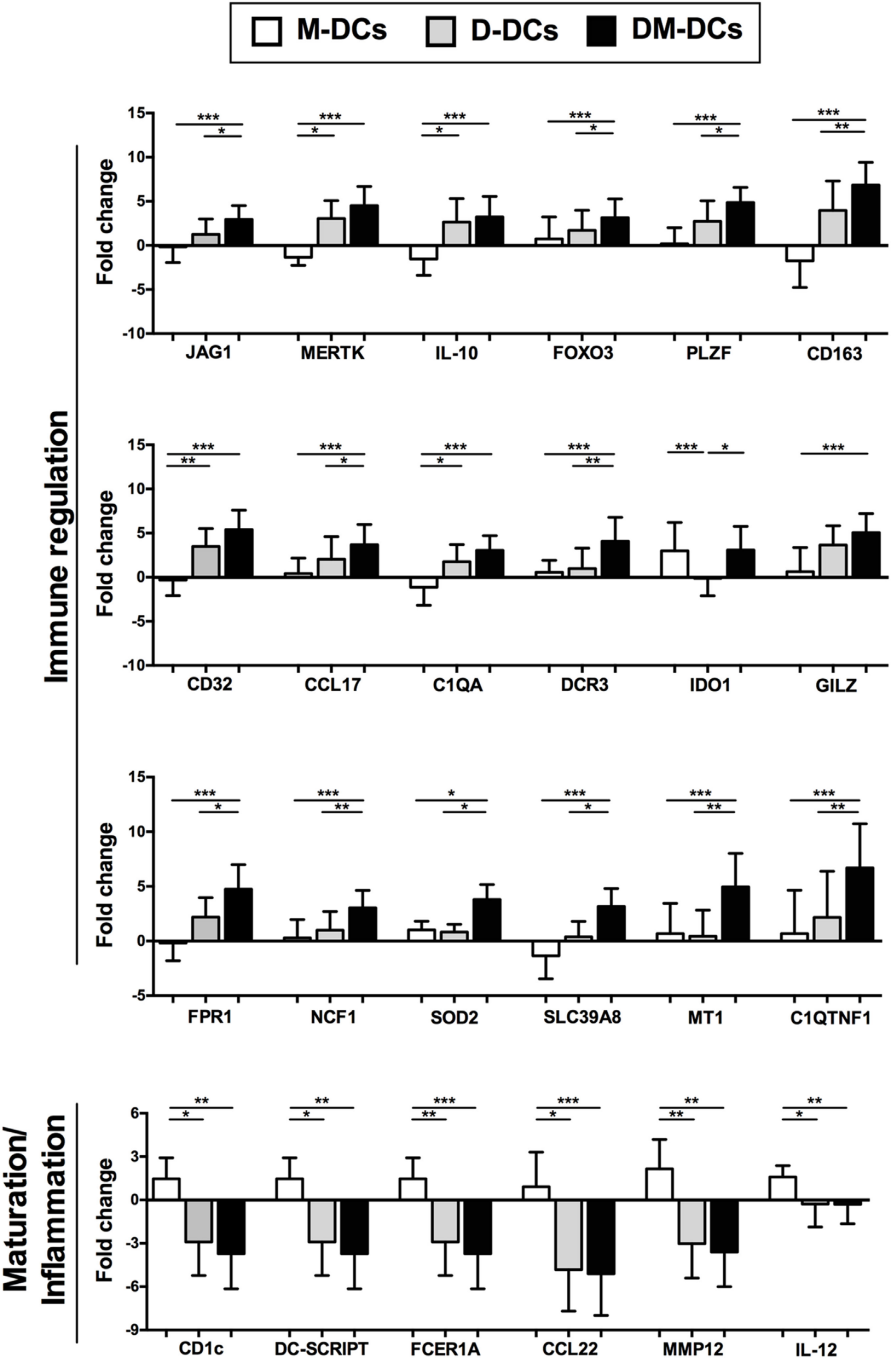
Dexamethasone-modulated and monophosphoryl lipid A (MPLA)-activated dendritic cells (DM-DCs) show upregulation of tolerance-related genes and downregulation of genes involved in maturation and inflammatory response. Gene expression levels of differentially expressed genes modulated by dexamethasone and MPLA in DM-DCs was confirmed by real time PCR. Results are shown as Fold Change values with respect to the untreated control (DCs). Data represent mean ± SD for 10 independent experiments. *p-value ≤ 0.05; **p-value ≤ 0.01; ***p-value ≤ 0.001.

**Figure 6:**
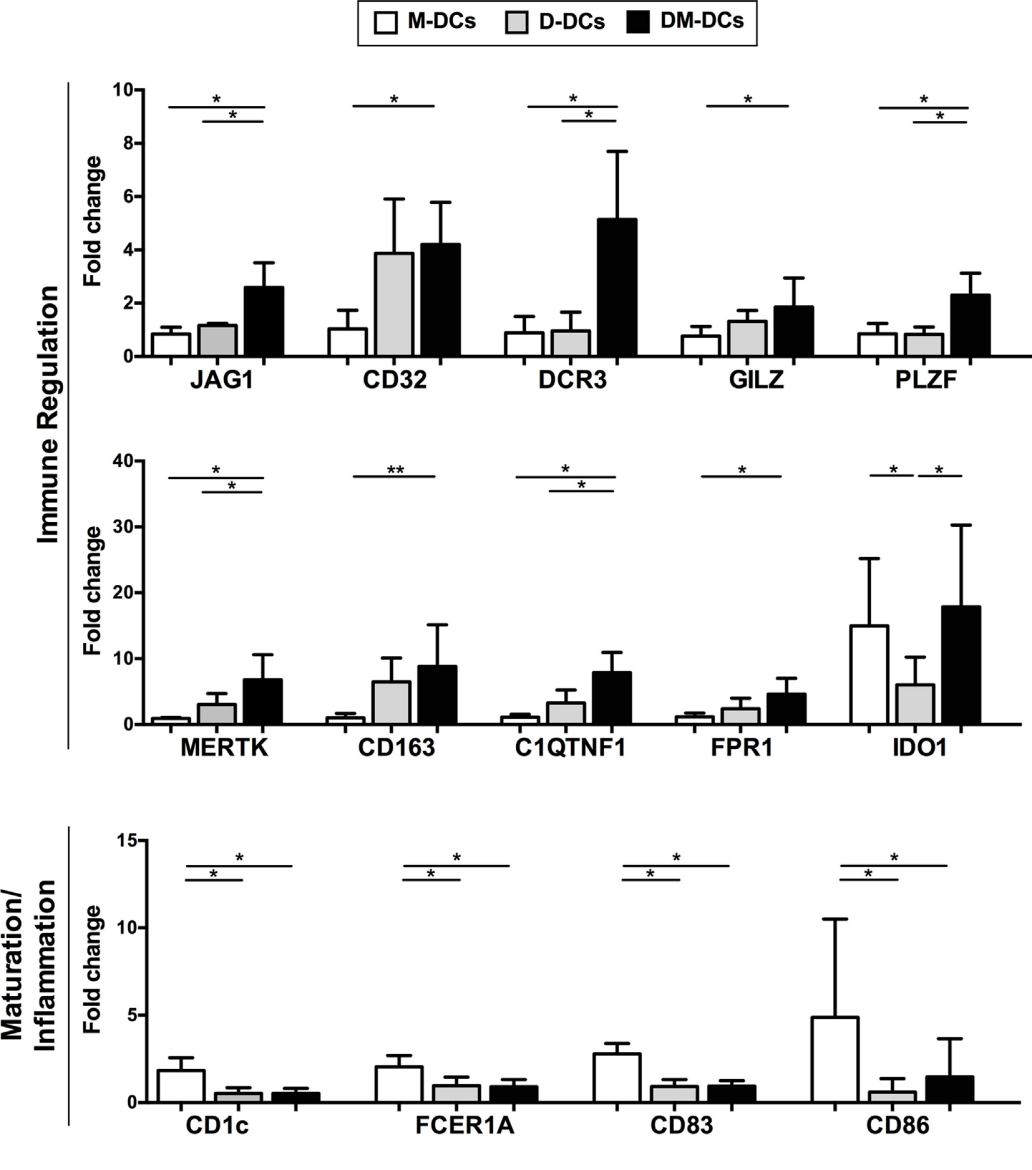
Changes in gene expression induced by dexamethasone and monophosphoryl lipid A are translated at the protein level. Protein levels of genes related with tolerance induction and monocyte-derived dendritic cells (DCs) activation were assessed by flow cytometry analysis. Results are shown as fold change values with respect to the untreated control (DCs). Data represent mean ± SD for five independent experiments. *p-value ≤ 0.05; **p-value ≤ 0.01.

The differential expression of several genes of interest that are modulated on DM-DCs such as CD163, JAG1, IDO1, MERTK, MT1F, FPR1, and CD32, which might participate in the main processes enriched on DM-DCs, was confirmed through real-time PCR (Figure 5) and were shown to correlate with protein levels determined by flow cytometry (Figure 6).

## Discussion

Here, we demonstrate, by whole-genome transcriptomic analysis of different moDC subtypes, that DM-DCs exhibit a distinctive transcriptional program that potentially endows them with regulatory functions through modulation of chemotactic responses, cell-to-cell signaling and interaction, and metabolic processes.

Glucocorticoids, including Dex, have been widely used for tolDC generation and have been demonstrated to inhibit DC maturation and inflammation (30, 31). Since activation of tolDCs has been shown to increase lymph node homing, antigen presentation, and stability against other inflammatory modulators (32), we activated Dex-treated DCs with MPLA (DM-DCs), a non-toxic clinical grade analog of LPS, which also signals via toll-like receptor 4 and exhibits potent immune-stimulatory capacity (33, 34). Glucocorticoid receptors are transcription factors that suppress the pro-inflammatory program induced by TLRs and in turn potentiate TLR-mediated anti-inflammatory responses such as IL-10 secretion (35, 36). Our group has previously shown that Dex and MPLA induce a tolerogenic profile in moDCs from both healthy controls and RA patients (21, 22). The resulting DM-DCs exhibit characteristic tolDCs features, with low expression of co-stimulatory and maturation markers, high production of anti-inflammatory cytokines, and reduced capability to promote effector T-cell responses. Consistent with their phenotypic properties, we found that modulation of moDCs with either stimulus alone or a combination of both also induces a distinctive transcriptional profile, which allows separation of moDCs samples according to differentially expressed genes.

Several methallothioneins (MTs) (MT1G, MT1H, MT1E, MT2A, MT1M, MT1A, and MT1X) appear in this study as some of the most upregulated genes within the 259 DE transcripts of DM-DCs. These are small cysteine-rich metal-binding proteins involved in the regulation of homeostasis of zinc and other heavy metals at cytoplasmic level (37). MTs can also affect different cellular processes such as gene expression, apoptosis, proliferation, and differentiation (38) and have been described to suppress collagen-induced arthritis via induction of TGF-β and reduction of pro-inflammatory modulators such as TNF (39) and to promote the expansion of Treg (40, 41). Besides MTs, another molecule involved in zinc transport, the zinc importer SLC39A8/ZIP8, is also highly upregulated in DM-DCs. Zinc is known to act as a modulator of immune responses through its availability, and zinc deficiency affects the immune system leading to increased inflammation and inflammatory diseases such as RA (38). In DCs, zinc supplementation has been shown to interfere with maturation, by inhibiting the upregulation of MHCII and co-stimulatory molecules (42), as well as to induce the expression of the tolerogenic markers PD-L1, IDO1, and CD103 (43). Thus, a tight regulation of zinc concentration is required and zinc may contribute to the immunoregulatory functions of DM-DCs, since several regulators of intracellular zinc concentration are overexpressed in these cells.

Cluster analysis of DE genes in DM-DCs (with respect to DCs) revealed six different expression patterns, highlighting genes associated with a single modulatory agent. Genes induced by MPLA were enriched in molecules involved in IFN signaling as well as DC differentiation and maturation-related genes, which were in turn downregulated by Dex treatment, confirming previous reports that Dex impairs DC maturation and induces an immature-like DC phenotype (31, 44). MPLA stimulation also induced the expression of the regulatory molecule IDO1, which was similar in DM-DCs and M-DCs. This finding was to be expected since IDO upregulation has already been described in mature DCs, in particular in TLR-stimulated DCs (45–47). It does, however, differ from the work of Danova et al. (48), whom reported a weak IDO expression in MPLA-treated DCs. Differences between both studies may be explained by differences in the generation protocols as well as the detection techniques used. Dex also induced several molecules associated with regulation of immune responses involving Treg and effector T-cell functions (JAG1, TBXAS1, and MERTK) (49, 50), DC differentiation and function (IRAK3, GILZ, C1Q, and STAB1) (28, 51, 52) and suppression of inflammatory signaling (MAP3K8/TPL-2, FCGR2B, and VDR). Functional enrichment analysis showed that Dex treatment of moDCs also induced the expression of genes related to the complement system pathway. Of great interest among these genes is C1Q, previously described to be upregulated in tolDCs and proposed as potential marker of tolerogenicity (28). C1Q has been demonstrated to be a potent modulator of DCs, which suppresses DC differentiation and activation through engagement of the inhibitory receptor leukocyte-associated Ig-like receptor 1, limiting the activation of immune responses (53, 54). Moreover, C1Q was shown to inhibit T-cell activation and pro-inflammatory cytokine production, while enhancing IL-10 secretion (55).

Additionally, we identified a group of genes that were induced by a synergistic effect of Dex and MPLA. These genes include anti-inflammatory mediators (SLC39A8/ZIP8, CCL18, and C1QTNF1/CTRP1) and molecules involved in the regulation of T-cell function (MT1, THBS1/TSP-1, and TNFRSF6B/DcR3). Interestingly, several DE transcripts within this cluster are associated with production of reactive oxygen and nitrogen species (FPR1, FPR2, NCF1, and SLAMF1), and accordingly, IPA analysis of our dataset predicted the activation of this biological function.

Furthermore, metabolic changes seemed to play an important role in DM-DCs, since processes associated with free radicals, in particular ROS production and fatty acid metabolism, were enriched in the DM-DCs dataset. Despite usually being considered pro-inflammatory, ROS participate in many physiological processes. Excessive ROS drives inflammation and oxidative damage, while low ROS amounts were shown to suppress immune responses (56, 57). Effector T cells exhibit impaired proliferation and increased apoptosis in response to sustained pro-oxidant conditions, whereas Treg is less sensitive to this effect and retain their suppressive function (58). Correspondingly, ROS production is one of the strategies used by Treg to suppress effector T cells (58, 59). Macrophages have been demonstrated to suppress T-cell responses by producing ROS and induce Treg in a ROS-dependent manner (60). Dex has been previously shown to increase ROS production in macrophages and moDCs (61). Here, we show that Dex treatment alone induces NCF1 and PDK4, which are both involved in ROS production and we demonstrate that MPLA activation after Dex-mediated modulation of moDCs leads to the upregulation of several genes involved in ROS metabolism and production. It has been previously described that modulation of moDCs is accompanied by changes in cellular metabolism and that tolDCs show a different metabolic profile than pro-inflammatory DC subsets (26). This catabolic and highly energetic metabolic profile of tolDCs may be due to higher energy demands required for suppressive functions (62). Therefore, in DM-DCs, regulation of ROS production and zinc homeostasis could be crucial to the regulatory function of DM-DCs.

Another hallmark of DM-DCs is the regulation of chemokine expression. In particular, the upregulation of Treg and naive T-cell attractants (CCL17, CCL18, CCL23/MIP-3, and CXCL9) and chemokines associated with the recruitment of monocytes and granulocytes (CCL13/MCP-4, CCL26, and CXCL5) as well as the downregulation of expression of chemokines attracting effector T cells might account for the potential to recruit Treg subsets to sites of inflammation to promote tolerance.

Of note, this is the first work investigating the molecular basis of tolerogenic features of moDCs modulated with Dex and alternatively activated with MPLA. We have demonstrated that besides inhibiting DC maturation and inflammation, Dex and MPLAs treatment jointly induce a distinctive transcriptional profile in moDCs mainly regulating pathways involving cellular chemotactic responses, cell-to-cell signaling and interaction, as well as zinc and ROS metabolism, favoring the recruitment and proliferation of Treg while inhibiting effector T-cell responses. Our results indicate that there is a broad spectrum of immunoregulatory properties of tolDCs beyond the already described mechanisms depending on direct DC–T-cell contact and anti-inflammatory cytokine secretion and thus provides novel targets for immunotherapeutic strategies based on tolDCs.

## Ethics Statement

All subjects gave written consent according to the Declaration of Helsinki, and all procedures were approved by the Ethics Committees of the Faculty of Medicine and the Clinical Hospital of University of Chile. Consent forms are held by the authors and are available for review by the Editor-in-Chief.

## Author Contributions

All authors read and approved the final version of the manuscript. JA and RV had full access to all the data from the study and take responsibility for the integrity of the information and the accuracy of the data analysis. PG-G and KS contributed equally. PG-G, KS, JA, RV, and RT participated in study conception and design. JM and ML were responsible of recruitment and sample collection of healthy subjects. PG-G, KS, AM, HN, JM, BP, and OA participated in data acquisition. PG-G prepared the manuscript. PG-G, KS, AS-G, RV, JA, DC, RT, MM, and DC participated in analysis and interpretation of data and manuscript revision.

## Conflict of Interest Statement

The authors declare that the research was conducted in the absence of any commercial or financial relationships that could be construed as a potential conflict of interest.

## Supplementary Material

Table S1 Demographic characterization of donors.

Table S2 Primer sequences of target genes.

Table S3 Differentially expressed (DE) genes found on DM-DCs.

Table S4 Functional and pathway analysis of DE genes found on DM-DCs.

Table S5 Functional and pathway analysis of DM-DCs genes from Cluster 6.
