# Supplementary material for: Dexamethasone and Monophosphoryl Lipid A Induce a Distinctive Profile on Monocyte-Derived Dendritic Cells through Transcriptional Modulation of Genes Associated With Essential Processes of the Immune Response
Source: Front Immunol. 2017 Oct 23;8:1350. doi: 10.3389/fimmu.2017.01350 (PMC5660598; doi:10.3389/fimmu.2017.01350)
Supplement: Supplementary file 6 [file Data_Sheet_1.DOCX]

Supplementary Material

**“Dexamethasone and monophosphoryl lipid A induce a distinctive profile on monocyte-derived dendritic cells through transcriptional modulation of genes associated with essential processes of the immune response”**

**Paulina A. García-González^1^**^,2,#^, Katina Schinnerling^1,2,#^, Alejandro Sepúlveda-Gutiérrez^3^, Jaxaira Maggi^1,2^, Ahmed M. Mehdi^4^, Hendrik J. Nel^4^, Bárbara Pesce^1^, Milton Larrondo^5^, Octavio Aravena^1^, María C. Molina^1^, Diego Catalán^1,2^, Ranjeny Thomas^4^, Ricardo A. Verdugo^3*^, Juan C. Aguillón^1,2*^

*Correspondence:

**Dr. Ricardo A. Verdugo**. [raverdugo@u.uchile.cl](mailto:raverdugo@u.uchile.cl)

**Dr. Juan C. Aguillón.** [jaguillo@med.uchile.cl](mailto:jaguillo@med.uchile.cl)

## Supplementary Figure 1


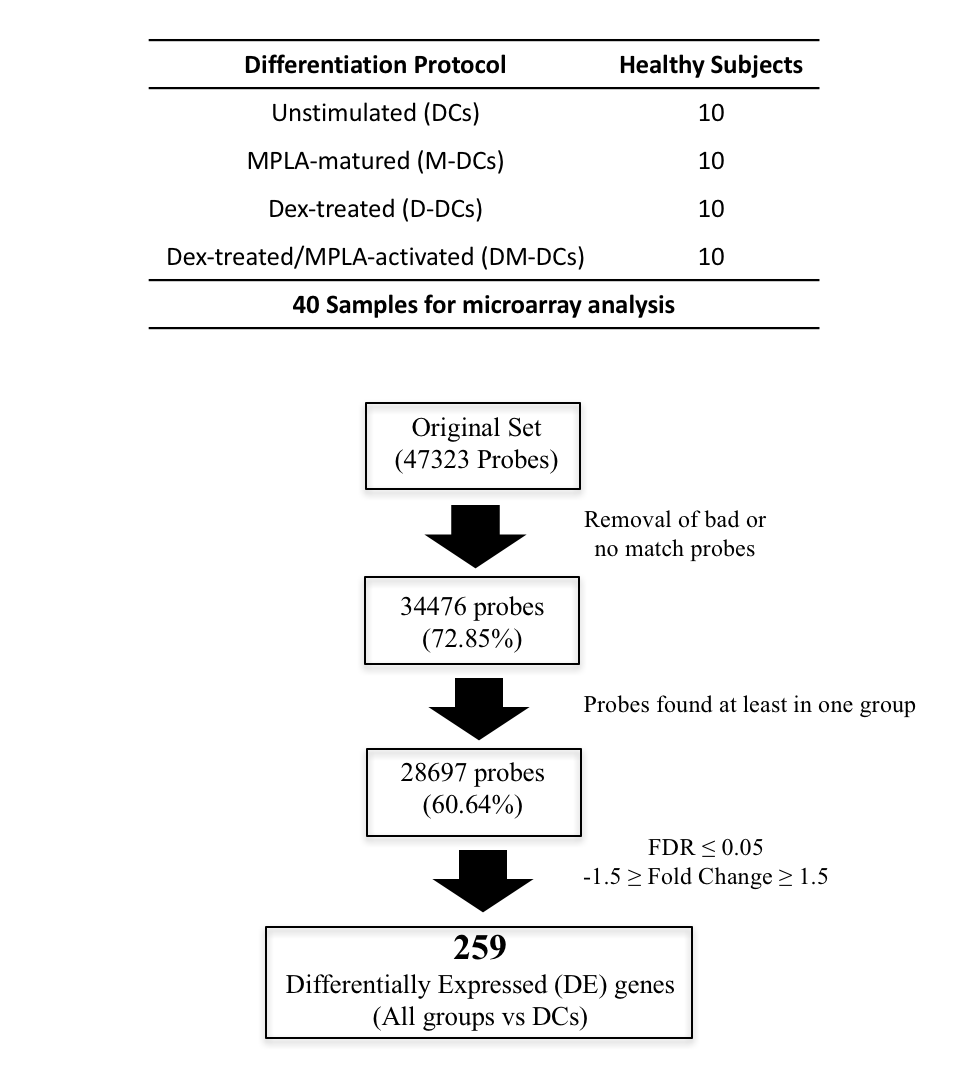


**Supplementary Figure 1.** Microarray analysis settings.

**Supplementary Figure 2.**


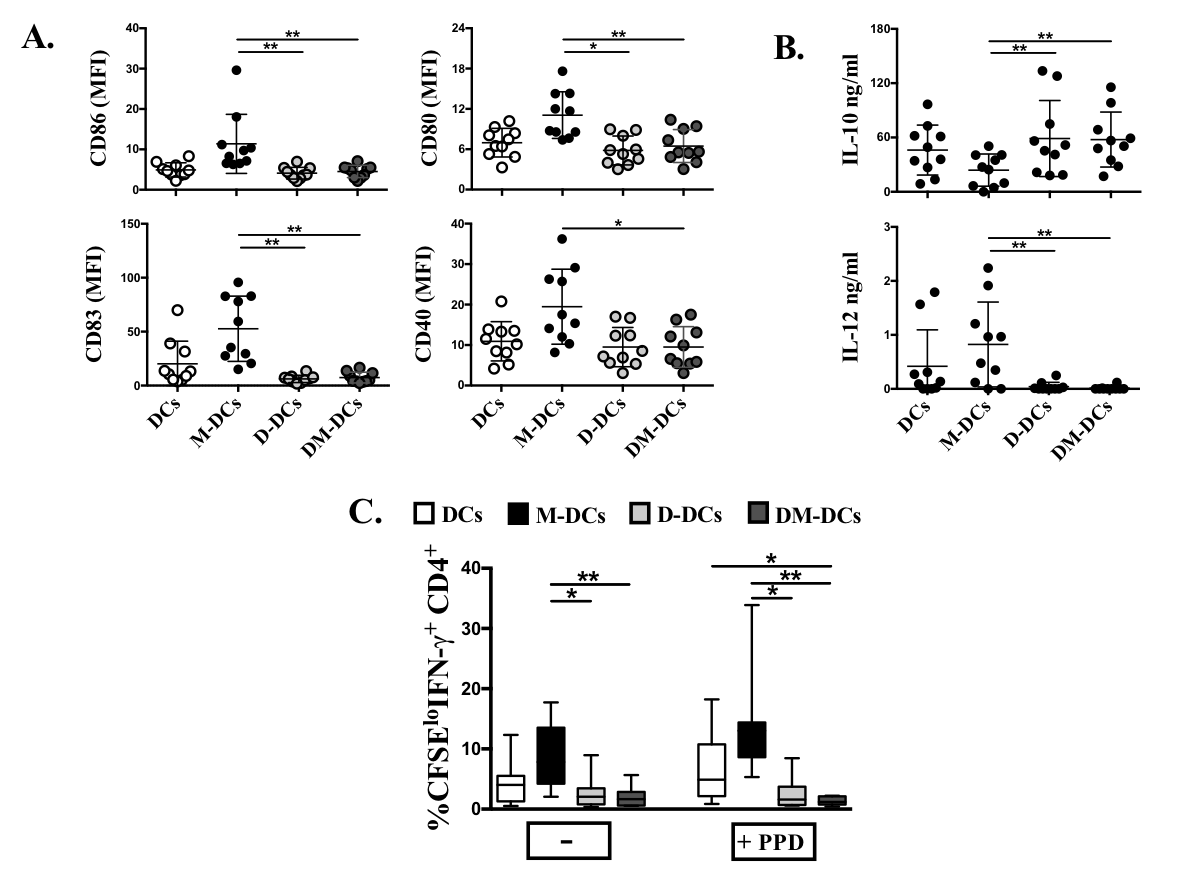


**Supplementary Figure 2.** Monocyte-derived DCs modulated with dexamethasone (Dex) and monophosphoryl lipid A (MPLA) exhibit tolerogenic phenotype and function. A, Expression of phenotypic markers was assessed by flow cytometry. B, Cytokine secretion in response to CD40L was determined by ELISA. C, Proliferation (by means of CFSE dilution) and intracellular IFN-gamma (IFN𝛄) were analized in cocultures of unpulsed or PPD-pulsed DCs and autologous CD4+ T cells. Data represent mean ± SD for 10 independent experiments. n=10. *p-value ≤0.05; **p-value ≤0.01. MFI, mean fluorescence intensity.
